# Mitigating COVID-19 Impact on the Portuguese Population Mental Health: The Opportunity That Lies in Digital Mental Health

**DOI:** 10.3389/fpubh.2020.553345

**Published:** 2020-11-16

**Authors:** Cristina Mendes-Santos, Gerhard Andersson, Elisabete Weiderpass, Rui Santana

**Affiliations:** ^1^Department of Culture and Society, Linköping University, Linköping, Sweden; ^2^NOVA National School of Public Health, Public Health Research Centre, Universidade NOVA de Lisboa, Lisbon, Portugal; ^3^Fraunhofer Center for Assistive Information and Communication Solutions, Porto, Portugal; ^4^Department of Behavioural Sciences and Learning, Linköping University, Linköping, Sweden; ^5^Psychiatry Section, Department of Clinical Neuroscience, Karolinska Institutet, Stockholm, Sweden; ^6^International Agency for Research on Cancer, Lyon, France

**Keywords:** COVID-19, public mental health, digital mental health, internet interventions, Portugal, EU, eHealth and eMental-health

## Abstract

COVID-19 mitigation measures present unprecedented challenges in mental healthcare delivery, posing high risk to the mental health of at-risk populations, namely patients diagnosed with COVID-19, frontline healthcare providers, and those submitted to quarantine or isolation measures, as well as the general population. Ensuring safe and equitable access to mental healthcare by these groups entails resorting to innovative psychosocial intervention strategies, such as digital mental health. In this perspective piece, we describe the impact of COVID-19 on the Portuguese population's mental health, present an overview on initiatives developed to address the challenges currently faced by the Portuguese mental healthcare system, and discuss how the timely implementation of a comprehensive digital mental health strategy, coupling research, education, implementation, and quality assessment initiatives, might buffer COVID-19's impact on the Portuguese society.

## Introduction

The COVID-19 pandemic is a major public health emergency of international concern (1). As of 13th August 2020, there have been 20,439,814 confirmed cases and 744,385 deaths worldwide, with 188 countries having reported at least 1 case (2). In Portugal, the first confirmed case was diagnosed at 2nd March 2020, and since then, the spread has been fast, contaminating 53,548 people and totalizing 1,770 deaths (3). Infected patients may present a wide range of symptoms, namely fever, cough, myalgia, fatigue, sputum production, headache, hemoptysis, diarrhea, and/or dyspnea (4). Most patients seem to present with a mild disease. However, possibly as many as 20% appear to progress to severe disease, including pneumonia, respiratory failure, and, in some cases, death (5).

Due to potentially serious health outcomes of COVID-19, draconian unprecedented mitigation and suppression measures have been taken by many countries to stop the spread of the virus (6). In Portugal, the government declared an emergency state in 18th March 2020 (7), and measures, such as canceling gatherings and events, closing schools, limiting the number of people in public places (e.g., supermarkets, pharmacies, etc.), recommending social isolation, and mandating telework whenever possible have been taken to reduce contact rates in the general population and reduce transmission. Regarding suspect and diagnosed cases, a range of measures have been adopted, such as early identification, contact tracing and monitoring, and prophylactic isolation or mandatory quarantines (8).

The implementation of such mitigation measures combined with insufficient preparedness of health authorities, high unpredictability of the outbreak itself, and uncertainty of its social-economic impact may lead to widespread fear, anxiety, and social alarm, posing high risk to the mental health of the Portuguese population (9).

### COVID-19 Impact on Mental Health

Literature on the impact of COVID-19 on mental health is still scarce. However, research on the emotional consequences of the current and previous outbreaks, such as severe acute respiratory syndrome (SARS), Middle East respiratory syndrome-related coronavirus (MERS), and Ebola virus disease indicates a high burden of mental health problems among patients, suspect cases and close contacts, frontline healthcare providers (10), those submitted to isolation and quarantine measures (11, 12), informal caregivers (10), the elderly (13), and the population at large (14). Prevalent mental health problems include depression, anxiety, psychological distress (10, 15), burnout, panic attacks, post-traumatic stress disorder (16, 17), and insomnia (12). Other adverse mental health outcomes frequently reported are fear, anger, stigmatization, low self-esteem, and lack of self-control (18, 19). Severe conditions, such as psychotic symptoms (12) and suicidality (20, 21) have also been reported, although less frequently.

Epidemiological data on the prevalence of COVID-19 precipitated mental health disorders in the Portuguese population are still limited. However, previous studies reported a high burden of mental health disorders in Portugal with estimated lifetime prevalence for at least one psychiatric disorder of 42.7%. When compared to other countries participating in the *World Mental Health Surveys Initiative*, lifetime prevalence for such disorders was only exceeded by the USA (47.4%). All other Western European countries had lower prevalence values, namely Spain (19.4%) and Italy (18.1%), figures that underline the vulnerability of the Portuguese population in this domain (22).

Ongoing research (9, 23) promoted by Escola Nacional de Saúde Pública—Universidade Nova de Lisboa, inquiring 157,927 respondents, highlights the potential catastrophic impact the actual pandemic might have in citizens' mental health. In that study, around 83% of participants reported low mood, feeling agitated, anxious, or sad due to physical distance measures 1 week after such measures were enforced. More than 26% reported feeling this way daily or almost every day. The youngest (16–25 years of age) and female respondents were the most susceptible to confinement measures-induced distress. In addition, a positive association has been identified between the perception of risk to contract COVID-19 and the frequency of reported adverse mental health outcomes, such as feeling anxious, agitated, down, or sad (9).

Concerning healthcare workers, so far, only one study addressed mental health. In that study (24), 76.7% of participants reported moderate to high levels of fatigue, and 68.8% of healthcare workers reported anxiety levels above normal, with physicians reporting the highest levels of anxiety.

No data are yet available on the pandemic's impact on COVID-19 Portuguese patients. However, previous research (25) acknowledges this group as an at-risk population.

### Populations at Risk

Patients diagnosed with COVID-19 and suspect cases may fear the outcomes of this possibly lethal disease experiencing anxiety, emotional distress, and insomnia (10). Potential stigmatization and social exclusion may spiral into other mental health conditions, such as adjustment disorders and depression (11). Additionally, symptoms' manifestation and treatment adverse effects may aggravate premorbid mental health disorders (10). Previous research has also found increased prevalence of post-traumatic stress disorder among survivors of infectious diseases (16).

Frontline healthcare providers are submitted to enormous pressure (24) due to a high risk of infection, potential scarcity of resources, and overwork. Such work conditions, aggravated by potential discrimination and lack of contact with support networks, make this group susceptible to complex emotional reactions and mental health problems, such as stress, anxiety, depressive symptoms, insomnia, burnout, traumatic stress, denial, anger, and fear (16, 26, 27). Reported risk factors (27–31) include being female, history of physiological chronic non-communicable diseases, family history of mental disorders, working at isolation wards, professions requiring close contact with infected patients, such as being a nurse or a medical technician, and having relatives with suspected or confirmed COVID-19. Of additional concern is the impact such conditions might have on healthcare providers' performance, potentially compromising the quality of healthcare, increasing the occurrence of medical errors and incidents, and ultimately hindering the fight against COVID-19 (31).

Special attention should also be provided to those submitted to quarantine or isolation measures. Confusion, boredom, loneliness, anxiety, anger, and guilt associated to the effects of contagion, quarantine, and stigma on family and friends are common experiences (12). Moreover, research on the psychological impact of quarantine in previous outbreaks found that being quarantined is a significant immediate and long-term risk factor to the mental health of both healthcare providers and the general population (12, 16). A study (32) targeting parents and children submitted to quarantine reported that mean post-traumatic stress scores were four times higher in children who had been quarantined than in those who were not quarantined, and almost 1/3 of quarantined parents in that study fulfilled diagnosis criteria of a trauma-related mental health disorder compared to 6% of parents who were not quarantined. Another study (33) focusing on Australian horse owners submitted to quarantine due to an equine influenza outbreak reported high psychological distress in this group when compared to the general population. Concerning healthcare providers, several studies attest the deleterious and long-term impact of quarantines on mental health outcomes (12, 16, 18). Having been quarantined has been identified as a predictor of depression (34) and post-traumatic stress symptoms (35) up to 3 years after the event and to be positively associated with alcohol abuse or dependency symptoms in healthcare workers (36). Another study reported quarantined staff were significantly more likely to report exhaustion, detachment from others, anxiety, irritability, insomnia, sub-optimal work performance, and absenteeism (37). Previously identified stressors comprise longer quarantine duration, infection fears, frustration, inadequate supplies, inadequate information, financial loss, and stigma (12).

Still to be assessed is the impact of COVID-19 global confinement measures on mental health. Nevertheless, recent research highlights the role social capital might have in improving quality of sleep and reducing anxiety in self-isolated individuals (14).

### Innovative Psychosocial Intervention Strategies

Considering the transversal and significant impact COVID-19 pandemic might have on the mental health of the general population and high-risk groups, immediate action must be taken to manage the imminent upsurge of mental health disorders associated or aggravated by coronavirus outbreak circumstances (38). Confinement measures should not enforce paralysis, and innovative psychosocial intervention strategies capable of preventing, screening, monitoring, and intervening at this level must be developed and implemented, ensuring safe and equitable access to mental healthcare (39). One such strategy is digital mental health.

Digital mental health is understood as the use of digital technologies (e.g., telephone, mobile devices, apps, videoconference and chat software, psychological assessment, support and intervention platforms, artificial intelligence, virtual reality, serious games, etc.) (40, 41) to support and improve mental health conditions and provide mental healthcare including screening, health promotion, prevention, early intervention, treatment, or relapse prevention (42). It encompasses a wide range of modalities that might be particularly suitable in this outbreak context, namely internet research (43, 44), screening and tracking tools (45, 46), videoconferencing counseling and psychotherapy (45, 46), internet interventions (38, 45, 47), and e-learning and e-supervision (48).

Facing COVID-19 mental health-imposed challenges requires a comprehensive strategy (25), where the abovementioned modalities are interlinked and prevention/intervention programs are adjuvated by high-quality training programs and research. In the following sections, we elaborate on how such modalities could be useful during the COVID-19 crisis and report on ongoing initiatives of this kind being developed in Portugal.

#### Internet Research

Conducting online behavioral and mental health research associated with the COVID-19 outbreak is key to gather information on the pandemic's impact on different target populations and deliver evidence-based tailored public health interventions (49).

In Portugal, important initiatives have been launched in this domain by Escola Nacional de Saúde Pública—Universidade Nova de Lisboa (9, 23), Instituto de Saúde Pública—Universidade do Porto (50), and CESOP—Universidade Católica Portuguesa (50, 51) to assess the general population and frontline healthcare providers' adaptation to the outbreak and mitigation measures.

Complementarily, the Portuguese Psychologists Association created a task force supporting the expedite assessment and dissemination of research projects aiming at identifying and monitoring the population's mental health unmet support care needs and assessing the efficacy and cost-effectiveness of prevention and intervention programs or healthcare models implemented during the COVID-19 pandemic. Seventy-six online questionnaire studies are ongoing under this umbrella initiative (52) focusing on topics ranging from the use of digital technologies by psychologists during the pandemic to the characterization of COVID-19's impact on general, specific, and clinical populations.

Surprisingly, none of these studies aims at studying the effects of COVID-19 on patients diagnosed with the disease or survivors. An immediate priority is, therefore, collecting high-quality data on COVID-19's short- and long-term impact on brain function, cognition, and mental health of patients with or recovering from COVID-19 (53).

Moreover, it is vital to perform implementation research, namely pragmatic clinical trials assessing the efficacy and cost-effectiveness of different digital mental health services implemented during this pandemic (e.g., based on videoconference, apps, chatbots, etc.), to support peri and future resource allocation decisions (54). Such initiatives should take into consideration digital health equity factors and involve people from marginalized and vulnerable groups in codesign during development and implementation (55). Tackling this challenge requires integration across disciplines and institutions, and new sources of funding (53).

In this regard, the Portuguese Foundation for Science and Technology has launched specific calls to promote research on COVID-19, namely Gender Research 4 COVID-19, Research4COVID-19, and AI 4 COVID-19 (56). Nevertheless, more funding is necessary to address digital mental health research gaps in this domain and incentivize the development or adaptation of innovative tools capable of preventing, diagnosing, and mitigating the population's distress during this outbreak.

#### Screening and Tracking Tools

The development or adaptation of screening and tracking tools to assess and monitor mental health outcomes in high-risk populations, such as COVID-19 patients, healthcare providers, and those in quarantine, could be particularly helpful during this crisis. Screening web platforms, apps, and chatbots are highly scalable and, if coupled with artificial intelligence, have the potential of identifying mental health pressing needs and referring or providing first-aid responses to at-risk subjects (57).

In this context, chatbots are particularly interesting due to their conversational workflow and easy and rapid deployment across email, web, social media, and text (58). During COVID-19 crisis and beyond, chatbots could be used to harness the healthcare system not only by screening and triaging citizens and healthcare providers at risk of developing mental health disorders but also by supporting in prompt education and referral.

Another interesting application of artificial intelligence in this domain is the monitorization of social networks to model pandemic trends as well as monitoring public reactions to the pandemic over time (59), facilitating psychological crisis interventions (49). Initiatives of this kind have already saved lives in China (60), and could be helpful in responding to digital native suicidal ideation since this appears to be one of the most vulnerable groups to confinement measures-induced distress (23).

Finally, leveraging all the above-mentioned dimensions, digital phenotyping is a promising strategy to passively monitor at-risk populations during crisis, such as the COVID-19 outbreak. Encompassing the passive collection and analysis of a range of behavioral data in mobile devices, including, but not limited to, spatial trajectories (via GPS), physical mobility patterns (via an accelerometer), social networks, social dynamics (via call and text logs and Bluetooth), and voice samples (via microphone) (61), digital phenotyping has the potential of increasing accuracy and bringing timeliness to the psychological assessment process (62).

To the best of our knowledge, initiatives of these kinds are not yet ongoing in Portugal, and mentioning such approaches in a country where digital mental health is at its infancy, such as Portugal, and during a crisis, may sound as pure science fiction and a waste of time. However, in technology, the future rapidly becomes the present and dissemination occurs fast, especially in times of urgency, such as the current moment. Since such approaches may be intrusive, conflict with individual freedoms, or leave vulnerable populations behind (59), their implementation must be carefully thought out and framed to guarantee that their development and implementation comply with ethical, legal, and cultural requirements and their integration in online or hybrid-healthcare models is assured, certifying that patients are adequately signaled and referred to online or physical psychiatric and psychological counseling/psychotherapy services.

#### Tele and Videoconference Counseling and Psychotherapy

Telephone and online psychological counseling/psychotherapy services are instrumental in providing immediate response to acute population needs and ensuring continuation of care and adequate follow-up of patients with pre-outbreak mental conditions (39).

In this regard, various helplines have been made available by hospitals, associations, and academic agencies to provide support during this crisis (63), and on the 1st of April 2020, a partnership between the Shared Services of the Portuguese Health Ministry, Calouste Gulbenkian Foundation, and the Portuguese Psychologists Association has launched a national counseling helpline to support the population (64). As of 20th July 2020, this helpline had already received 23,590 calls from healthcare providers and the general population (65), highlighting the importance of providing such first-aid resources to contain the population's distress.

Considering psychological counseling/psychotherapy services, an abrupt shift to this treatment modality has occurred after enforcement of mitigation measures, and on the 7th April 2020, the Portuguese Psychologists Association officially published Guidelines for the Provision of Psychology Services Mediated by Information and Communication Technologies (66), recommending its use during this crisis. From 4th May 2020 onwards, the Portuguese deconfinement plan started to be implemented, and clinical activity in hospitals and private practice was progressively resumed. The real number of tele and video consultations performed by psychologists and psychiatrists during and after the confinement period is not available for consultation. Yet, an analysis of available data from the Lisbon Psychiatric Hospital Centre, assumed here as a proxy, reveals a 37% decrease in telemedicine appointments in June 2020 (post-confinement) when compared to April 2020 (during confinement) (67), suggesting that a full return to the traditional face-to-face model is unlikely, and a hybrid mental healthcare model will probably emerge from this crisis. Awareness about such treatment options, patients' preferences, potential changes in providers' attitudes (68), and digital mental health research on the effectiveness and cost-effectiveness of these modalities might facilitate ongoing integration of technology (69) in the Portuguese mental healthcare system.

#### Internet Interventions

With millions of citizens confined or complying with social isolation recommendations worldwide (70) and, therefore, at risk not only of developing mental health conditions but also at increased risk of inactivity (71) and malnutrition (72), wider public digital health approaches may also be necessary to deliver health promotion and intervention programs (38). In this regard, internet interventions—self-help guided or unguided interventions based on established psychotherapy models operated via secure platforms or mobile apps that aim at providing synchronous or asynchronous health and mental health–related assistance (73, 74)—may play a pivotal role in increasing the availability of self-care psychoeducational content and delivering evidence-based psychological intervention protocols (14).

Internet interventions have been found to be more effective than treatment as usual or as effective as face-to-face therapies for most COVID-19 triggered mental disorders, namely depression (75–78), generalized anxiety disorder (79–81), panic disorder (82, 83), insomnia (84), and post-traumatic stress disorder (85). Additionally, growing evidence endorses its beneficial impact in supporting patients with somatic conditions, such as chronic pain (86), tinnitus (87), irritable bowel syndrome (88), diabetes (89, 90), and cancer (91–94).

Due to its high flexibility, adaptability, dissemination potential, and low delivery costs (74), internet interventions seem to be a viable approach to effectively support the general population as well as at-risk and vulnerable groups, such as chronic patients now deprived of routine healthcare (95). The equitable implementation of self-guided, guided, or blended approaches, possibly following a stepped care model, would facilitate psychoeducation delivery, contribute to citizens' empowerment, and ease the burden over healthcare providers, allowing them to focus on patients with severe conditions, ultimately contributing to the resilience of the healthcare system.

However, only a handful of such programs were under development or ongoing in Portugal [e.g., (96–99)] prior to the COVID-19 crisis, and, to the best of our knowledge, very few internet-delivered initiatives were developed/adapted to address COVID-19 specific constraints in the meantime (e.g., internet-delivered multimodal pre-habilitation program for confined cancer patients) (100), suggesting that well-known implementation barriers, namely clinicians' attitudes and lack of knowledge, training, and experience, persist (68). Such barriers are probably compromising the development, adaptation, and implementation of internet interventions during this crisis in Portugal.

#### Comprehensive e-Learning and e-Supervision Initiatives

While the COVID-19 crisis may be operating as a catalyst effect on the wide-scale acceptance and adoption of digital mental health initiatives (38), attitudinal and training barriers (68) must be expeditiously addressed in Portugal, or significant digital mental health strategies will remain unexplored, resulting in costly missed opportunities to the Portuguese mental healthcare system and its users. Overcoming such barriers implies developing and delivering adequate e-learning and e-supervision programs capable of mitigating the lag between a psychologist's instruction and unfolding practice.

In this respect, initiatives under development in Portugal, such as webinars (101) and professional guidelines (66) are important but clearly insufficient. Portuguese universities and associations must take the lead and develop comprehensive (on- and off-the-job) training initiatives capable of fulfilling clinicians' immediate education needs and practical concerns. Equipping the workforce with such cost-effective strategies will not only provide the necessary tools to handle the COVID-19 crisis but also enable facing the second mental health crisis that will loom in the following months, with economic recession (102).

Furthermore, digital mental health must become part of psychology courses' syllabus, and curricular and professional internships in this domain must be organized to train future clinicians in the development, refinement, and implementation of high-quality digital mental health tools and interventions.

Nevertheless, such reform is easier to imagine than to implement. Most Portuguese universities are not prepared to introduce such adjustment in their curriculums, and most faculty members hold classical stances and education, not being prepared to train future clinicians to work within a digital paradigm. Mapping and bringing together national clinicians and researchers working in the field and partnering with leading international organizations with expertise in delivering digital mental health programs might be an important contribution to achieve this goal.

### Paving the Road Toward a Digital Mental Healthcare Paradigm Shift

Shifting to a digital mental healthcare paradigm entails more than willingness from clinicians, researchers, or academics. The involvement of other digital mental health ecosystem stakeholders—patients/citizens, charities and associations, companies, funders, and policymakers—is crucial to guarantee the alignment between digital mental health policy, regulatory and quality assurance frameworks, and citizens' interests.

In November 2019, an important step toward this unfolding digital revolution was taken with the publication of the National Strategic Telehealth Plan (103). Aggregating contributions from a wide range of stakeholders—members of central and regional healthcare administrations and professional nursing, medical, and pharmacists' associations—this plan aims at identifying the main challenges the country faces in this domain and proposing strategic measures to expedite the full integration of telehealth within the everyday sphere of healthcare.

Surprisingly, mental health is not mentioned in this document, and the misrepresentation of the Portuguese psychologists' association as an institutional stakeholder may be an important red flag suggesting that, once again, policymakers' attention might have focused on healthcare priorities other than mental health. It may also be the case that this omission reflects the void of initiatives ongoing in the country pre-outbreak, denouncing the embryonic stage that characterizes digital mental health in Portugal, and explaining the limited digital mental health resources applied so far to face the consequences of COVID-19 crisis. In fact, despite decades of significant evidence on the efficacy and cost-effectiveness of digital mental health initiatives worldwide (104–107), the National Mental Health Plan (108) fails to acknowledge the potential of digital mental health in contributing to promote the mental health of the Portuguese population and providing access to timely mental healthcare.

COVID-19 may have the potential of introducing disruption into the status quo. It may have the positive unintended effect of moving the Portuguese healthcare system forward by exposing its limitations and demanding a call for action. However, for this side effect to unfold, digital mental health must be recognized as a strategic opportunity not only to mitigate COVID-19's impact on the Portuguese population mental health but also to promote it beyond this pandemic. Chasing rainbows is not an option in this or the following mental health crisis. The solution lies on rethinking the National Mental Health Plan (108) at the light of the digital paradigm; aligning it with the National Strategic Telehealth Plan (103); delineating a comprehensive operational plan capable of leveraging duly funded training and implementation research initiatives; and ensuring the digital mental health road starts being paved today, with strategic implementation.

## Conclusion

In summary, acknowledging digital mental health as a tactical opportunity and investing in a comprehensive digital mental health plan, coupling research, education, implementation, and quality assessment initiatives, will buffer COVID-19's impact on the Portuguese society, particularly in high-risk groups. By promoting resilience in the population and preventing the upsurge or aggravation of mental disorders, digital mental health will also strengthen the already severely burdened Portuguese mental healthcare system (22), making it capable of facing future challenges more effectively.

## Data Availability Statement

The original contributions presented in the study are included in the article/supplementary material, further inquiries can be directed to the corresponding author/s.

## Author Contributions

All authors listed have made a substantial, direct and intellectual contribution to the work, and approved it for publication.

## Conflict of Interest

The authors declare that the research was conducted in the absence of any commercial or financial relationships that could be construed as a potential conflict of interest.
